# Prognostic value of molecular events from negative surgical margin of non-small-cell lung cancer

**DOI:** 10.18632/oncotarget.10949

**Published:** 2016-07-29

**Authors:** Bangrong Cao, Lin Feng, Dan Lu, Yan Liu, Yu Liu, Suping Guo, Naijun Han, Xiangyang Liu, Yousheng Mao, Jie He, Shujun Cheng, Yanning Gao, Kaitai Zhang

**Affiliations:** ^1^ State Key Laboratory of Molecular Oncology, Department of Etiology and Carcinogenesis, Cancer Institute & Hospital, Peking Union Medical College and Chinese Academy of Medical Sciences, Beijing, China; ^2^ Department of Basic Research, Sichuan Cancer Hospital & Institute, Chengdu, Sichuan Province, China; ^3^ Department of Thoracic Surgical Oncology, Cancer Institute & Hospital, Peking Union Medical College and Chinese Academy of Medical Sciences, Beijing, China

**Keywords:** negative surgical margin, epithelial-to-mesenchymal transition, prognosis, non-small-cell lung cancer, gene-expression signature

## Abstract

It is hypothesized that the molecular status in negative surgical margin (NSM) is associated with prognosis of cancer patients. In this study, the prognostic relevance of Epithelial-to-Mesenchymal Transition (EMT) molecular events in NSMs in patients with NSCLC was investigated. EMT model was developed, in which the mesenchymal transition of human immortalized bronchial epithelial cell line was induced by TGF-beta1. Gene expression of EMT-induced cells and NSMs from 60 lung squamous cell carcinoma (SCC) patients was profiled by microarray and validated by quantitative RT-PCR. Two independent cohorts (lung SCC, *n* = 50; NSCLC, *n* = 54) were employed to validate the prognostic value of candidate genes. A set of 1490 genes were identified in EMT model *in vitro*. An EMT-like gene-expression pattern by 33 essential genes was optimized in NSMs, and was significantly associated with tumor progression. The 33 genes also exhibited a site-dependent field cancerization effect in the normal-appearing airways adjacent to NSCLCs. In the independent lung SCC cohort, the EMT-like active pattern indicated poor outcome of patients (*n* = 50, log-rank *p* = 0.009). Furthermore, in the NSCLC cohort, patients with EMT-like active pattern had shorter predictive survival time (*n* = 54, log-rank *p* = 0.02). In conclusion, the existence of EMT-like gene expression in NSMs, may play critical role in tumor progression and be a potential biomarker for prognosis in patients with NSCLC.

## INTRODUCTION

Lung cancer is the leading cause of cancer-related deaths worldwide [1, 2], with a major morphologic subtype (nearly 80% of all cases) of Non-small-cell lung cancer (NSCLC). Surgical resection is one of the most effective treatments for NSCLC, especially for the early stage diseases. Unfortunately, it was reported that approximately 30-40% of stage I NSCLC patients would die of cancer after curative surgical resection of tumor [3, 4], which is a major clinical issue for early disease.

Securing cancer-free surgical margin (or negative surgical margin, NSM) is a critical goal for surgeons in curative surgical treatment of lung cancer. The traditional method to confirm a clean surgical resection is based on pathological diagnosis on margins of normal tissue. Whereas, according to previous large cohort studies, the microscopic residual (R1) margin rate following pulmonary resection of lung cancer was proximately 3-7% [5–7]. R1 margins adversely affect the outcomes, with a decline in 5-year overall survival from 62% to 37% in patients with stage I diseases [7]. On the other hand, about 20% of patients with neither microscopic nor macroscopic cancer residuals (R0 resection) at surgical margins would suffer local recurrence after surgery [8]. Thus, the traditional pathological assessment of NSM is insufficient to predict outcome of NSCLC. A more precise approach to evaluate the resection margins is in a great need to identify patients at high risk for more effective adjuvant therapy.

In recent years, accumulated evidences have demonstrated genetic, epigenetic or transcriptional alterations in microscopically normal-appearing tissues adjacent to cancers in head, neck, colon, rectum, prostate, breast, lung, liver, esophagus, stomach, and skin et al [9–11], that is referred as the “field effect” of cancerization. TP53 gene mutations [12], methylation at promoters of a series of genes [13], gene expression profiles [14] in non-malignant airways were proposed as biomarkers for early detection of lung cancer. Moreover, K-ras mutation at codon 12 in NSMs was detected and significantly associated with local recurrence of NSCLC [15]. The concept of field cancerization suggests that the malignant molecular changes emerge long before the morphological alteration, and they could serve as “molecular margins” in the assessment of surgical margins of lung cancer.

Epithelial-to-Mesenchymal Transition (EMT) confers malignant traits on tumor cells, such as motility, invasiveness, and survival ability [16–18]. Extracellular signals from the tumor cell itself or the microenvironment of the tumor stroma are proposed to induce cancer cells to undergo EMT, which is the critical step for cancer metastasis and would indicate poor prognosis of patients. However, it is still unclear whether the EMT-related events can serve as biomarkers in molecular assessment of NSMs of NSCLC.

In this study, we identified an EMT-related gene-expression profile using an EMT model of cultured non-malignant bronchial cell line *in vitro*. This profile was further analyzed in NSMs from 60 cases with lung squamous cell carcinoma (SCC) *in vivo* by microarray and real time PCR. Prognostic evaluation of this gene-expression subtype was performed in two independent cohorts of NSCLC.

## RESULTS

### EMT of immortalized bronchial epithelial cells induced by TGF-β1

**Figure 1:**
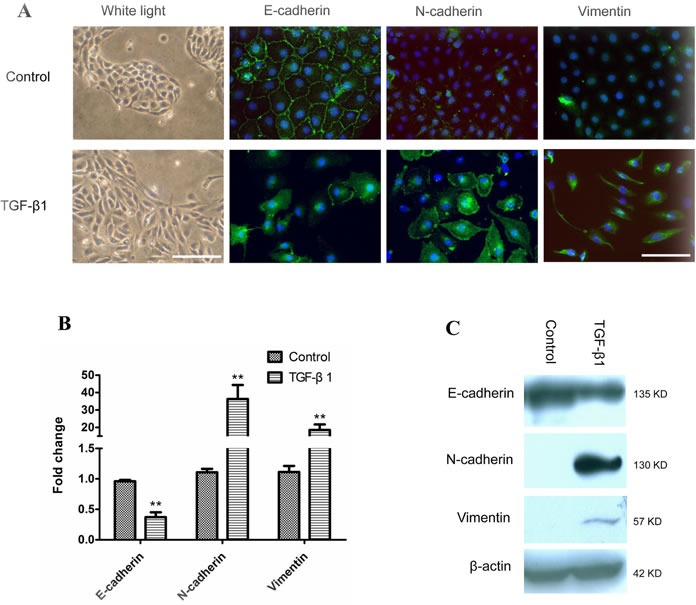
EMT of human immortalized bronchial epithelial cells induced by TGF-β1 M-BE cells were treated with human recombinant TGF-β1 at a final concentration of 5 ng/ml for 6 days, with cells cultured without TGF-β1 as control. **A.** A phenotypic change in M-BE from epithelial to spindle-shaped morphology was observed after TGF-β1 treatment, which was photographed at 100× magnification using white light microscopy (left panel, scale bar = 100 μm). Immunofluorescence staining showed the expression status of three EMT markers (left to right panels: E-cadherin, N-cadherin, Vimentin) in M-BE cells induced by TGF-β1. FITC (green) was used for respective target proteins; 4,6-diamidino-2-phenylindole (DAPI) was used to visualize nuclei. All of the fluorescence images were captured at 400× magnification using fluorescence microscopy (scale bar = 25 μm). **B.** qRT-PCR analysis for mRNA levels of three EMT markers in TGF-β1 treated cells. Y-axis indicates the relative expression level (Fold Change, FC) of genes. Means and standard deviations (SD, error bars) are shown. Unpaired Student's t-test (two sided) was performed for significance estimate. **M-BE cells treated with TGF-β1vs control, *P* < 0.05. **C.** Western blotting shows the protein expression levels of E-cadherin, N-cadherin and Vimentin. β-actin is presented for equivalent loading control.

After being treated with 5 ng/ml of TGF-β1 for six days, the morphology of M-BE cells was dramatically changed from a normal epithelial phenotype to spindle-shaped phenotype with the loss of cell-to-cell contact (Figure 1A). In contrast with the control, TGF-β1-induced cells had a significantly elevated cytoplasmic expression of the mesenchymal markers N-cadherin and Vimentin, and dislocation of the epithelial marker E-cadherin from the membrane to the cytoplasm (Figure 1A). At mRNA levels, the expression of E-cadherin was significantly (*P* = 0.01) reduced in TGF-β1-treated M-BE cells compared with the control, while the N-cadherin (*P* = 0.05) and Vimentin (*P* = 0.03) were up-regulated significantly (Figure 1B). Meanwhile, the E-cadherin protein level was decreased, while the N-cadherin and Vimentin protein levels were elevated in the TGF-β1-treated M-BE cells (Figure 1C). These observations indicated that TGF-β1 successfully induced EMT in immortalized bronchial epithelial cells.

### Gene expression profiling of the EMT cell model

Clustering analysis based on global genes revealed that TGF-induced M-BE cells were strongly associated with an alteration in the transcriptome (Supplementary Figure S2A). Next, 2628 genes were identified as significant EMT associated genes (Supplementary Figure S2B, Supplementary Table S3), including 1490 up-regulated genes (FC > 2, FDR < 0.01) and 1138 down-regulated genes (FC < 0.5, FDR < 0.01) in EMT-induced cells. Those up-regulated genes were mostly related to cell adhesion, actin cytoskeleton organization, cell motion/migration, vasculature development and wound healing (Supplementary Figure S2C, Supplementary Table S4).

### Activation of EMT-related genes in NSMs and being correlated with tumor progression

To investigate whether the EMT-related genes identified *in vitro* were activated in NSMs *in vivo*, gene expression profiling was performed on NSMs from 60 primary lung SCC patients. Gene Set Enrichment Analysis (GSEA) showed that the EMT up-regulated genes were associated with TNM stage (Table 1, NES = 1.39, FWER *p*-value = 0.08). Further analysis indicated that this association was mainly restricted to pathological N stage (Supplementary Figure S3A, Table 1, NES = 1.71, FWER *p*-value < 0.001), but not T stage (Table 1, NES = 1.05, FWER *p*-value = 0.45). However, the other clinical parameters (such as gender, age, smoking index, or tumor differentiation) showed no statistical significance with EMT up-regulated genes. It was interesting that the EMT down-regulated gene set did not achieve statistical significance in this analysis with any clinical phenotype, including N stage (Supplementary Figure S3B, NES = 0.95, FWER *p*-value = 0.61).

**Table 1:** Association analysis between clinical parameters and EMT-related gene sets by GSEA

	EMT up-regulated			EMT down-regulated		
Phenotypes^1^	NES	NOM *p*-val	FWER *p*-val^2^	NES	NOM *p*-val	FWER *p*-val
Gender(Male *vs* Female)	−1.223	0.17	0.209	−1.133	0.247	0.348
Age(≥ 60 ys *vs* < 60 ys)	1.059	0.35	0.432	−1.128	0.258	0.331
Smoking(≥ 20 pys *vs* < 20 pys)	−1.196	0.198	0.231	−0.949	0.549	0.609
T(T3&T4 *vs* T1&T2)	1.054	0.394	0.448	0.729	0.94	0.949
N(N1&N2 *vs* N0)	1.705	<0.001	<0.001***	0.95	0.552	0.612
TNM Stage(I&II *vs* III)	1.386	0.068	0.08*	0.664	0.975	0.976
Differ.(P *vs* M&W)	0.928	0.539	0.648	−1.246	0.142	0.2

^1^ ys, years; pys, package years; Differ., tumor differentiation grade; P, Poor; M, Moderate; W, Well. ^2^ *, FWER *p* value < 0.1; ***, FWER *p* value < 0.001.

### Common gene-expression features between metastatic genes derived from NSMs and EMT-related genes

**Figure 2:**
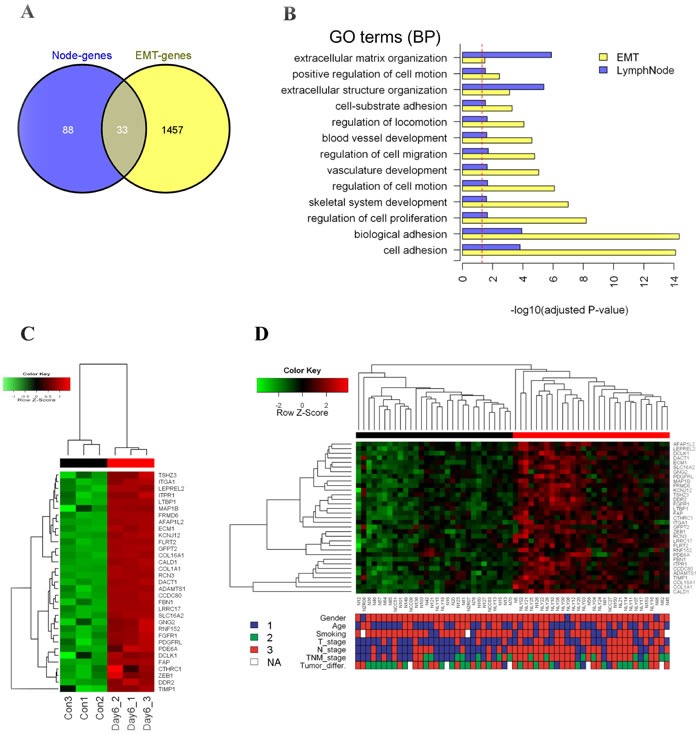
Identification of EMT-related genes in NSMs of lung SCC with lymph node metastasis **A.** Venn diagram showing the overlapped 33 genes between EMT model (EMT-genes) and lymph node derived genes (Node-genes). **B.** Overlapped significant GO BP terms between EMT-genes and Node-genes in (A). X-axis indicates -log_10_ transformed Benjamini-Hochberg adjusted *P*-value. Dash line indicates significant cutoff (adjusted *P*-value = 0.05). **C.** Unsupervised hierarchical clustering analysis of the 33 genes on the EMT model dataset (red bars indicate EMT-active cells, black for the control). The colored matrix indicates the relative expression level of genes (red for higher expression, green for lower, the same to D). **D.** Unsupervised hierarchical clustering analysis of the 33 genes on the NSMs dataset (red cluster with up-regulated pattern of 33-gene was defined as active cluster, while the down-regulated was defined as inactive cluster). The down panel shows the corresponding clinical parameters of patients, and compared with two gene-expression clusters: Gender (1: female, 3: male, Fisher's exact test *P*-value = 1); Age (1: < 60 years, 3: ≥ 60 years, chi-square test *P*-value = 0.79); Smoking (1: <20 package years, 3: ≥ 20 package years, chi-square test *P*-value = 0.068); T_Stage (1: T1&T2, 3: T3&T4, chi-square test *P*-value = 0.03); N_Stage (1: N0, 3: N1&N2, chi-square test *P*-value = 0.0007); TNM_Stage (1: I, 2: II, 3: III, chi-square test *P*-value = 0.0007); Tumor_differ. (tumor differentiation, 1: well, 2: Moderate, 3: poor, chi-square test *P*-value = 0.38).

Significance Analysis of Microarrays (SAM) identified 121 lymph node metastasis associated genes (Supplementary Figure S4A, delta = 0.75, FDR = 0.069, and Supplementary Table S5) in the lung SCC dataset. Interestingly, all of the significant genes were up-regulated in the lymph node positive samples. For these genes, most of the significantly enriched biological GO terms overlapped with those of the 1490 EMT up-regulated genes, such as extracellular structure organization, cell adhesion, regulation of cell motion/migration, and vessel development (Figure 2B, Supplementary Figure S4B, Supplementary Table S6).

Comparing the lymph node metastatic gene set and the EMT gene set, there were 33 common genes (Figure 2A, Supplementary Table S7), which were significantly overlapped (hypergeometric test, *P* < 0.01). The *in vitro* EMT model (Figure 2C) and the *in vivo* NSMs samples (Figure 2D) exhibited similar clustering patterns based on 33 common genes. The sample sub-group in which genes high expressed was referred to as EMT-like active pattern. Briefly, the EMT-active pattern was significantly correlated with positive lymph node (chi-square test, *P* < 0.01) and higher TNM stage (chi-square test, *P* < 0.01). In addition, lower T stage was enriched in the EMT-inactive group (chi-square test, *P* = 0.03). There was no significant association between EMT-active cluster and gender, age, smoking index, or tumor differentiation grade. Furthermore, GSEA performed on the 33 common genes showed similar results of correlation with the clinical parameters (Supplementary Table S8).

### Site-dependent field cancerization effect of the EMT-NSMs gene features in airways adjacent to NSCLC

**Figure 3:**
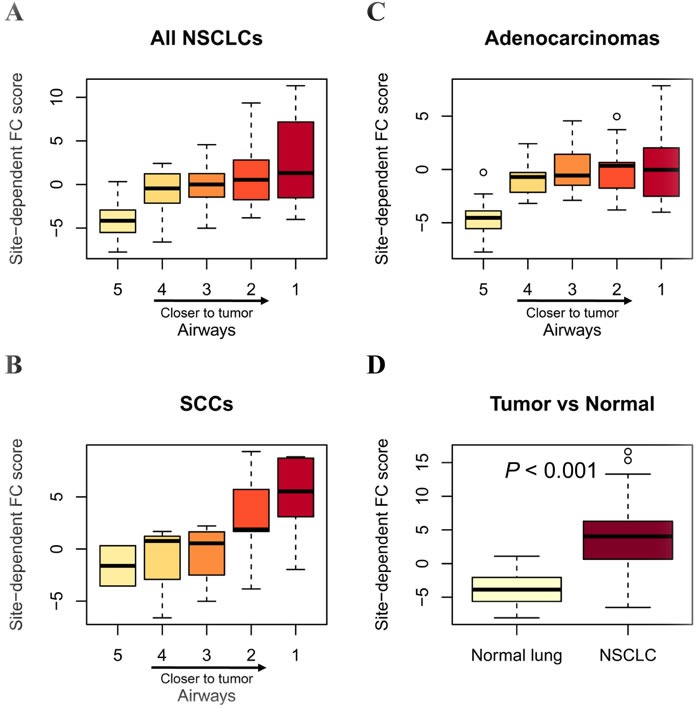
Site-dependent field cancerization effect of EMT-related genes in airways with respect to tumor proximity The site-dependent field cancerization (FC) score was quantified as described in Methods. Box plots depicting site-pendent FC score in airways from all NSCLCs (**A**), SCCs only (**B**) and Adenocarcinomas only (**C**), as well as the corresponding paired NSCLCs and normal lung tissues (**D**). Statistical analysis in (D) was performed by one-sided t tests. Heavy lines indicate medians, and whiskers indicate maximum and minimum FC scores. Airway distance from tumors is numerically indicated with a range of 1 (closest) to 5 (farthest).

We examined the gene expression profile of normal lung tissues, tumor tissues, as well as the corresponding airway brushing samples with various tumor proximities from 23 patients suffering NSCLC [19]. Of the EMT-related 33 genes, 32 ones were mapped to the airway dataset, and were used to estimate the site-dependent FC effect of the adjacent airways or tumor tissues as previously described [19]. Results showed that the site-dependent FC score were gradually increased in airways along with the shorter distance from tumors (Figure 3A), with a more pronounced site-dependent FC effect in lung SCCs (Figure 3B) than in Adenocarcinomas (Figure 3C). As expected, the site-dependent FC score was significantly elevated in tumors than in the adjacent normal lungs (Figure 3D). These findings suggest that the EMT-related features showed a dominant site-dependent FC effect in airways of NSCLC patients.

### Confirmation of EMT-related genes that were activated in NSMs from lung SCC patients

Of the 33 genes, four genes were selected according to their biological functions in EMT, and were further confirmed by qRT-PCR. As a result, *FBN1*, *ECM1*, *MAP1B*, and *LTBP1* were significantly elevated in EMT-induced M-BE cells (Supplementary Figure S5 A: Fold Change = 5.01, *P* = 0.02; Fold Change = 6.45, *P* = 0.02; Fold Change = 2.21, *P* = 0.04; Fold Change = 1.42, *P* = 0.04; respectively). In the NSMs of lung SCC, the four genes were significantly up-regulated in the lymph node positive samples (Supplementary Figure S5 B: *FBN1*, Fold Change = 2.75, *P* < 0.01; *ECM1*, Fold Change = 1.63, *P* < 0.01; *MAP1B*, Fold Change = 4.26, *P* < 0.01; *LTBP1*, Fold Change = 2.01, *P* < 0.01), compared with the lymph node negative samples. Moreover, we also revealed that all of the 4 genes were up-regulated in EMT-induced NSCLC cell line A549 (Supplementary Figure S6). In addition, the protein level of *ECM1* was confirmed in NSMs by IHC, and tended to be correlated with lymph node status (Supplementary Figure S7).

### Prognostic association of EMT-like gene-expression pattern in NSCLC patients

**Figure 4:**
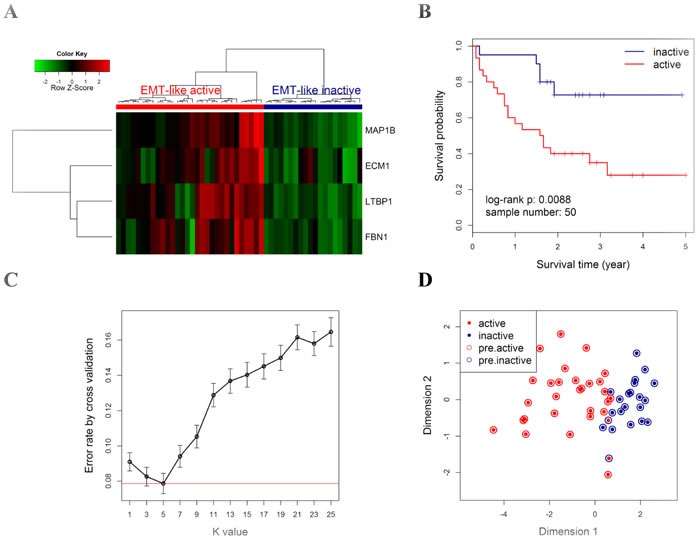
Prognostic value of EMT-like gene-expression pattern in the CICAMS cohort **A.** All patients (*N* = 50) were classified into two major groups (upper panel: red cluster, active; blue cluster, inactive) by unsupervised hierarchical clustering with the Euclidean distance and ward linkage method. The colored matrix indicates the relative expression levels of 4 genes by qPCR (red for higher expression, green for lower). **B.** Kaplan-Meier curves and log-rank tests were performed to compare the overall survival rates of the two groups of cases described in A. **C.** A 5-fold cross validation to select the best k value in kNN modeling for class prediction. For a series of k values (x axis), 5-fold cross validation was performed by 1000 random repeats, the mean (with 95% CI, error bars) of error rate is indicated (y axis). Red line indicates the lowest error rate (0.078) with k value = 5. **D.** Scatter plot for kNN training (k = 5) results of CICAMS dataset. All samples (each point) were mapped into a 2-dimension map by classical multidimensional scaling using the Euclidean distance. Solid points indicate the actual subtype, with red indicating the active pattern (active) and blue for the inactive pattern (inactive). Circles indicate the predicted subtype by kNN model, with red indicating the predicted active pattern (pre.active) and blue for the predicted inactive pattern (pre.inactive).

To evaluate the prognostic value of the four EMT-related genes in NSMs, qRT-PCR assay was applied on the validation cohort from CICAMS. Hierarchical clustering analysis showed that all patients were grouped into two major clusters, one of which exhibited an EMT-like active gene-expression pattern (Figure 4A). Furthermore, the patients with EMT-like active pattern had worse overall survival (Figure 4B, *n* = 50, log-rank *P* = 0.009) than those without. Multivariate Cox proportional hazards regression model adjusted by patient gender, age, TNM stage, and tumor differentiation grade showed that the EMT-like subtype was an independent prognostic factor (Supplementary Table S9, HR = 3.7, 95% CI = 1.2∼10.8, *P* = 0.02).

**Figure 5:**
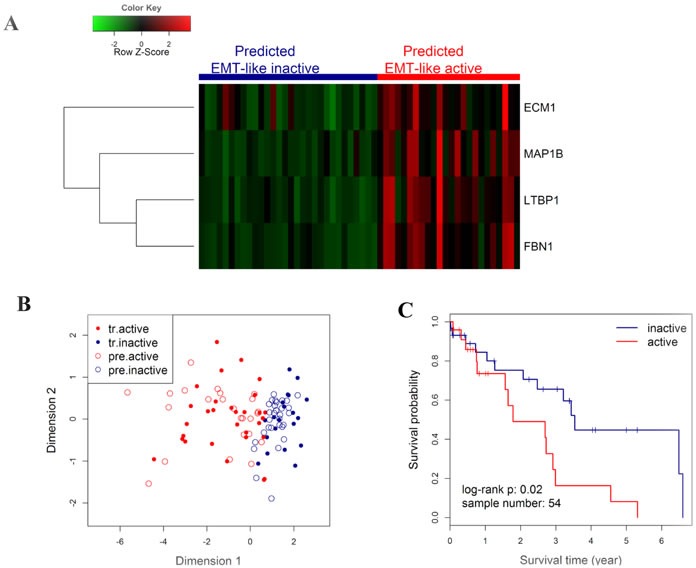
Outcome prediction in an independent NSCLC cohort from TCGA by EMT-like kNN predictor **A.** Heatmap showing the expression level of the 4 genes in the TCGA cohort, with colored bars on the top panel indicating the EMT-like status of each individuals as predicted in B. The colored matrix indicates the relative expression levels of 4 genes by RNA-seq (red for higher expression, green for lower). **B.** Scatter plot for kNN predicted (k = 5) results. Patients from TCGA were predicted by kNN classification model using the CICAMS cohort as training set. The 2-dimension scatter plotting indicates all samples by classical multidimensional scaling using Euclidean distance. Solid points indicate the training samples in CICAMS cohort, with red indicating the training active pattern (tr.active) and blue for the training inactive pattern (tr.inactive). Circles indicate the test samples in TCGA cohort, with red indicating the predicted active pattern (pre.active) and blue for the predicted inactive pattern (pre.inactive). **C.** Kaplan-Meier curves and log-rank test were performed to compare the overall survival rates of patients with different EMT-like status predicted in B.

Next, a kNN prediction model was trained in the CICAMS cohort and then tested in the TCGA cohort. In the training process, 5-fold cross validation revealed that the lowest overall error rate in the prediction of EMT-like subtypes was 0.07 (95% CI: 0.04∼0.10) when k value was 5 (Figure 4C). Only two samples were incorrectly classified by kNN model (k = 5) in the training dataset (Figure 4D). In the independent prediction process, 44.4% (24/54) of patients from TCGA cohort were predicted as EMT-like active gene-expression pattern (Figure 5A-5B), and they would have significantly worse overall survival (Figure 5C, *n* = 54, log-rank *P* = 0.02). Moreover, the association between EMT-like subtype and patient outcome was independent of gender, age, TNM stage, and the pathological type of patients (Supplementary Table S10, HR = 2.5, 95% CI = 1.0∼6.1, *P* < 0.05).

## DISCUSSION

In the present study, a set of EMT-related genes were identified in a respiratory epithelium-derived nonmalignant cell line M-BE induced by TGF-β1 *in vitro*. These genes showed active pattern in malignance-free surgical margins from lung SCC and were significantly associated with node metastasis. Four EMT-related genes were submitted to validation analysis and showed strong prognostic value for NSCLC.

Many studies have demonstrated that EMT contributes to malignant traits of cancer cells such as motility, invasiveness, or drug resistance [17, 18]. However, the potential roles of EMT in premalignant cells have rarely been studied. In this study, a human bronchial nonmalignant cell line exhibited mesenchymal characteristics with a scattered spindle-shaped morphology when treated with TGF-β1. Consistent with the cellular phenotypic changes, genes well-defined as mesenchymal markers, such as N-cadherin, Vimentin, *ZEB1* and Snail [20, 21], were activated in the transformed cells (Supplementary Table S3). E-cadherin is another important marker in EMT process. Although it was not significantly decreased in the TGF-β1-induced cells in this study, its cellular translocation from membrane to cytoplasm may also indicate the activation of EMT [22, 23]. Compared with another EMT model of the NSCLC cell line A549, the M-BE model shared 334 (hypergeometric test, *P* < 0.001) common up-regulated genes and 138 (hypergeometric test, *P* < 0.001) common down-regulated genes (Supplementary Figure S8). These results consistently support the EMT-derived gene signature in at least two cell lines.

In NSCLC, the field effect of cancerization is probably derived from the widespread molecular damages throughout the respiratory epitheliums by carcinogens exposure [10]. The histologically normal-appearing lung tissues or the bronchial epithelium adjacent to neoplastic lesions were detected with genetic alterations [12, 24, 25], epigenetic abnormalities [13], and gene-expression significance [14]. These data suggest that the molecular alterations in the nonmalignant tissues around the cancerous regions may play a critical role in the development of lung cancer. While, our results proposed that assessment of these molecularly malignant events in surgical margin as a potential “molecular margin” will be helpful in outcome prediction of NSCLC.

Seike et al. [26] found that the gene expression of 15 cytokines in the benign tissues adjacent to lung adenocarcinoma were associated with lymph node status, and were related to patient survival when combined with the gene expression patterns in tumor tissues. Our results were carried out only in NSMs from NSCLC, which suggested that the prognostic value of field effect could be independent of the molecular features of the tumor itself. Moreover, despite the Seike's results suggesting the inflammatory genes may act in the NSMs [26], much more details on the complex biological process were poorly understood. By integrating analysis with an induced EMT model *in vitro*, the present results partly explained that the molecular abnormalities in the NSMs from NSCLC were related with EMT, which also play malignant roles in cancer cells.

However, for NSMs, why and where did the EMT signal come from have been unclear. One possible explanation is that the molecular events observed in the tumor-adjacent tissues reflect the host response to maintain homeostasis, activating pathways such as the wound healing response, which is important for tissue repair and associated with EMT [27]. An alternative mechanism is that cancer cells may spread malignant signals to tumor microenvironment and surrounding and/or distant microenvironment, by secretion of cytokines such as TGF-β, TNF-α and *VEGFA* [28]. Further analysis on the molecular mechanisms of these current findings is warranted.

In the validation process, we selected four genes out of the 33 common genes mainly based on their potential biological meanings in EMT. This method was described as a biased approach for feature selection in signature development previously [29], that would provide much more details of the molecular mechanism and proposed to be more reproducible across different datasets. *FBN1* encodes one of the fibrillin family proteins, which were structural components extracellular microfibrils and play roles in TGF-beta activation and bioavailability [30, 31]. Extracellular Matrix Protein 1 (*ECM1*) is a major component of the extracellular matrix, would stimulate the proliferation of endothelial cells and promotes angiogenesis and cancer progression [32]. *LTBP1* mediates proTGF-β1 localizing to the extracellular fibrillin microfibrils to form latent complexes without biological activity, and participates in subsequent TGF-β1 activation by integrins or other factors [33, 34]. *MAP1B* interacts with dynamic microtubule network and regulates its assembly, polymerization and stabilizing [35, 36], which may play important roles in morphology change and migration/invasion phenotype when cell occurring EMT. However, the potential roles of the four genes in NSCLC were poorly reported in previous studies. It is reported that the gene expression of *ECM1* was significant associated with poor overall survival of lung ADC [37]. Tessema et.al reported that methylation of *MAP1B* promoter was more frequent in lung tumors with chronic obstructive pulmonary disease (COPD) than those without COPD [38].

Pathological lymph node stage is an effective prognostic factor for NSCLC [39]. Although the EMT-related gene signature was originated from node status, its prognostic value for NSCLC was independent of TNM stage. This is partly because the anatomic location and the number of resected lymph nodes may affect the accuracy of lymph node staging [40–42]. In addition, the histopathological examination of resected lymph node samples may also be underestimated for approximately 30% of positive nodes [43]. This indicates that detection of the EMT-related signature in NSMs from surgical resected samples, or biopsies may be a useful approach to identify tumor micrometastasis of lung cancer without pathological lymph node metastasis.

Local recurrence or metastasis was generally believed to account for the failure of therapy and poor prognosis of NSCLC [44]. Among the patients with stage I disease, nearly 30-35% of them will suffer relapse after initial surgical resection [1, 45], despite the histological confirmation of tumor-free surgical margin. While, the molecular status of the “normal” appearing lung tissues in the surgical margin and its clinical significance was rarely examined in NSCLC. According to our materials, the EMT-like gene-expression pattern in the NSMs was proposed to be a poor prognostic factor for NSCLC. One limitation is that the relapse information of patients analyzed in this study was not available, thus the predictive value for local recurrence of NSCLC of this gene-expression signature needs to be validated in further studies.

In summary, an EMT-like gene-expression subtype discovered in NSMs was associated with lymph node metastasis and overall survival of NSCLC patients. The EMT-related molecular events that reflect malignant behavior of cancer cells may predate the emergence of morphological changes in epithelial cells in the affected field of cancerization, making it a possible tool for outcome prediction for NSCLC patients in the future.

## MATERIALS AND METHODS

### Patients and tissue samples

NSMs were collected from lung SCC patients who had undergone surgical resection at Cancer Institute & Hospital, Chinese Academy of Medical Sciences (CICAMS) between 2009 and 2012. Patients with any of the following points were excluded: a) those received neo-adjuvant chemotherapy/radiotherapy; b) with positive surgical margins by pathology; c) with poor RNA quality, RIN (RNA integrity number) less than 6.5; d) without follow-up information of overall survival (for the validation cohort). Finally, 110 patients (60 in discovering cohort, and 50 in validation cohort) were involved in this study. NSMs were mainly about 3-5 cm distance from the primary tumor, and checked without visible cancer cells by histopathology (Supplementary Figure S1). Fresh tissues were treated with RNAlater^TM^ (Ambion, Austin, TX, USA) to prevent RNAs from degradation and then stored at −80°C before subsequent molecular analysis. Histological and clinical TNM stage information were classified according to the 2004 World Health Organization (WHO) classification. There were no statistically significant differences in clinical variables between discovering cohort and validation cohort (Supplementary Table S1). The use of human tissue samples for this study were reviewed and approved by the Ethics Committee of CICAMS (approval number: CH-BMS-014), with written informed consent from all patients.

One publicly available cohort of NSCLC (*n* = 54, composed of 17 lung SCC and 37 lung adenocarcinoma) patients from The Cancer Genome Atlas (TCGA) database were involved for independent validation. The clinical parameters of this cohort were summarized in Supplementary Table S2.

### Cell culture and TGF-β1 treatment

A human immortalized bronchial epithelial cell line (M-BE) was previously established and maintained in our laboratory [46]. Cells were cultured in serum-free LHC-9 medium, and incubated at 37°C with 3.5% CO_2_ [46]. M-BE cells in cultures were treated with human recombinant TGF-β1 (R&D System, Minneapolis, MN, USA) at a final concentration of 5 ng/ml for six days. Cells cultured without TGF-β1 were set as controls.

### Immunofluorescence staining and western blotting for EMT markers

Expression and cellular localization of three EMT markers (E-cadherin, N-cadherin and Vimentin) in the M-BE model were examined by immunofluorescence microscopy [47]. For western blotting, total cell lysate was extracted from M-BE cells with RIPA buffer (Pierce, Rockford, IL, USA), separated by electrophoresis on 10% SDS-PAGE gels, electrophoretically transferred onto a PVDF membrane, and examined for three EMT markers and β*-*actin.

### Quantitative RT-PCR analysis

RNA isolation and reverse transcription were performed using TRIzol^®^ and SuperScript^®^ II (Invitrogen, Carlsbad, CA, USA), respectively. For M-BE samples, qRT-PCR analysis was performed using the SYBR^®^ Green (Takara, Otsu, Shiga, Japan) method. For human tissue samples, the TaqMan^®^ (Applied Biosystems, Foster City, CA, USA) method was employed for the qRT-PCR analysis of 4-gene profile.

### Microarray gene-expression profiling and data processing of public dataset

All of the sample-labeling, hybridization, washing and scanning steps were conducted following the manufacturer's specifications [48]. Data extraction and annotation were performed using GeneSpring v 7.3.1 (Agilent, Pal Alto, CA). Normalization and probe selection were carried out using ‘limma’ package in R software. All the microarray raw data are publicly available in the Gene Expression Omnibus (GSE40374, GSE40588).

The normalized gene-expression profiles of normal lung tissues, adjacent airways and tumors were downloaded from Gene Expression Omnibus of series GSE44077. Gene feature matching between our dataset and the downloaded dataset was performed using Entrez Gene identifiers. The estimation of side-dependent field cancerization effect of airways was calculated with the method as described by Kadara *et.al* [19].

The normalized gene-expression data (Reads Per Kilobase per Million reads, RPKM) of RNA sequencing of NSMs from NSCLC as well as the corresponding clinical data were downloaded from TCGA data portal. The log_2_-transformed RPKM value was used for subsequent prediction analysis.

### Statistical analysis

Gene Ontology (GO) enrichment analysis for gene sets was performed using DAVID online functional annotation tool [49, 50]. All of the other statistic analysis in this study was performed using R software (http://www.r-project.org). More details of statistical analysis are available in the Supplementary Materials and Methods.

## SUPPLEMENTARY MATERIAL FIGURES AND TABLES




