# Supplementary material for: Prognostic value of molecular events from negative surgical margin of non-small-cell lung cancer
Source: Oncotarget. 2016 Jul 29;8(32):53642–53. doi: 10.18632/oncotarget.10949 (PMC5581137; doi:10.18632/oncotarget.10949)
Supplement: Supplementary file 1 [file oncotarget-08-53642-s001.pdf]

Prognostic value of molecular events from negative surgical margin of non-small-cell lung cancer

Supplementary Material

Immunofluorescence Staining for EMT Model

Expression of EMT markers in M-BE was analyzed by immunofluorescence microscopy. Cells were disseminated onto collagen-coated glass coverslips and fixed in 4% paraformaldehyde dissolved in phosphate buffered saline (PBS). The glass coverslips were rinsed three times with PBS, permeabilized with 0.5% Triton X-100 for 15 min, rinsed three times with PBS, incubated with 3% bovine serum albumin (BSA) in PBS supplemented with 0.1% Tween-20 for 30 min, and then stained for the expression markers with primary antibodies: rabbit anti-E-cadherin (Santa Cruz, Santa Cruz, CA, USA; 1:100 dilution), mouse anti-N-cadherin (BD Biosciences, San Jose, CA, USA; 1:150 dilution), and mouse anti-vimentin (Santa Cruz, Santa Cruz, CA, USA; 1:150 dilution) and respective secondary antibody IgG conjugated with FITC (Jackson ImmunoResearch, West Grove, PA, USA; 1:150 dilution). Cells were co-stained with 4,6-diamidino-2-phenylindole (DAPI) to visualize nuclei, mounted with fluorescent mounting medium and viewed by immunofluorescence microscopy.

Hematoxylin and eosin (H&E) and Immunohistochemistry (IHC) Staining for NSMs of lung SCC

Formalin-fixed and paraffin-embedded tissues were used for cutting 5-μm-thick sections. Sections were deparaffinized, ethanol-rehydrated, and stained with H&E for microscopic examination. IHC was performed using above sections as described previously [1]. In brief, the deparaffinized, ethanol-rehydrated sections were incubated with corresponding primary antibodies and biotinylated secondary antibody, then stain with HRP-DAB system, followed by nuclei counterstaining with hematoxylin. The primary antibody was rabbit anti-ECM1 (Abcam, ab126629, 1:100 dilution).

Western Blotting Analysis for EMT Markers

M-BE cells treated with or without 5 ng/ml TGF-β1for six days were harvested. Total cell lysates were prepared by lysis with RIPA buffer (Pierce, Rockford, IL, USA) in the presence of protease inhibitors. Samples containing 40 μg of total protein were electrophoresed on 10% SDS-PAGE and electrophoretically transferred onto a PVDF membrane. Nonspecific binding to the

membrane was blocked 30 minutes at room temperature with 5% nonfat milk at the dilution specified by the manufacturers. Membranes were later probed with different primary antibodies as indicated overnight at 4 ^{o}C. The membranes were washed for 10 minutes three times in PBS with 0.1% Tween-20 and then incubated by horseradish peroxidase-conjugated mouse or rabbit secondary antibodies (Jackson ImmunoResearch, West Grove, PA, USA; 1:2000 dilution) for 1 hour. The membranes were washed three times for 10 minutes in PBS with 0.1% Tween 20, and the antibody reactivity was visualized with the SuperSignal West Pico Chemiluminescent Substrate (Pierce, Rockford). The following antibodies were used in the analysis: rabbit anti-E-cadherin (Santa Cruz, Santa Cruz, CA, USA; 1:3000 dilution), mouse anti-N-cadherin (BD Biosciences, San Jose, CA, USA; 1:4000 dilution), mouse anti-vimentin (Santa Cruz, Santa Cruz, CA, USA; 1:1000 dilution) and *β*-actin (Sigma-Aldrich, St Louis, MO, USA; 1:2000 dilution).

Quantitative RT-PCR Analysis

Total RNA from cell line or tissue samples was isolated using the TRIzol® (Invitrogen, Carlsbad, CA, USA) method. 1 μg of RNA was treated with RNase-free DNase (Promega, Madison, WI), then converted into cDNA using SuperScript® II and the accompanying standard protocol (Invitrogen, Carlsbad, CA, USA). For the M-BE samples: Quantitative RT-PCR analysis was performed using the SYBR® Green (Takara, Otsu, Shiga, Japan) method; Cycle thresholds (Ct) greater than 35 were set to 35; ribosomal 18S rRNA was employed as endogenous control; all primers (Supplementary Table S11) were synthesized in Sangon Biotech (Shanghai) Co., Ltd.; one of the triple control M-BE samples was used as reference sample for the fold change (FC) calculation. For the human tissue samples: TaqMan® (Applied Biosystems, Foster City, CA, USA) probes and primers (Supplementary Table S12) of the selected 4 genes were applied for qRT-PCR analysis; cycle thresholds (Ct) greater than 40 were set to 40; RPLP0 and GUSB were employed as an endogenous control as previously described [2]; a RNA sample mixture from ten cases was used as reference sample for FC calculation. All qRT-PCR experiments were performed on the on the Mx3005P® QPCR System (Agilent, Pal Alto, CA). Average cycle threshold (Ct) of the triplicate experiments for each sample was used for the subsequent analysis. The gene expression was calculated using the $2^{-\Delta\Delta Ct}$ method [3], where $\Delta Ct = Ct_{target\ gene} - Ct_{endogenous}$, and $\Delta\Delta Ct = \Delta Ct_{individual\ sample} - \Delta Ct_{reference\ sample}$.

Whole Genome Gene Expression Microarray Analysis and Data Processing

Total RNA was isolated from M-BE cells and human tissues using TRIzol® reagent (Invitrogen, Carlsbad, CA), and then purified using the RNeasy® Mini Kit(Qiagen, Germantown, MD), according to manufacturer's instructions. RNA was quantitated using a ND-1000 UV-VIS Spectrophotometer (NanoDrop Technologies, Wilmington, DE), and the integrity of the RNA was assessed with the RNA 6000 Labchip kit in combination with the Agilent 2100 Bioanalyzer (Agilent, Pal Alto, CA). RNA samples which had 260/280 ratio above 1.8 and RIN (RNA integrity number) greater than 6.5 were used in subsequent microarray experiment.

All of the sample-labeling, hybridization, washing and scanning steps were conducted at our laboratory, following manufacturer's specifications [4]. Briefly, Cy3 labeled cRNA was generated from 500 ng input purified RNA by in vitro transcription using Agilent's Low RNA Input Linear Amplification Kit PLUS (Agilent, Pal Alto, CA). Then, 1.65 μg cRNA from each labeling reaction was hybridized to the Agilent Whole Human Genome Oligo Microarray (Agilent, Pal Alto, CA), at 65 ^{o}C for 17h. After hybridization, the slides were washed and then scanned with the Agilent G2505B Microarray Scanner System (Agilent, Pal Alto, CA). The fluorescence intensities on scanned images were extracted and preprocessed by Agilent Feature Extraction Software (v9.1).

Log_2-transformed data and annotations of all probes were extracted from the GeneSpring v 7.3.1 (Agilent, Pal Alto, CA), normalization was performed by median-absolute scaling method with the limma package of R software. For the Entrez gene ids which were mapped by two or more probes, the probe which had the largest mean fluorescence intensity across all samples was selected.

Data Processing of Gene Expression Profile of A549

The raw data ("cel" files of Affymetrix Human Genome U133 Plus 2.0 microarrays) of TGF-β1-induced EMT model of A549 cell line were downloaded from GEO of series GSE17708. Gene expression profiles were normalized with "RMA" method by R package "affy", and probe annotation was performed based on "hgu133plus2.db" from Bioconductor. Gene feature matching between our dataset and the downloaded dataset was performed using Entrez Gene identifiers. For the Entrez gene ids which were mapped by two or more probes, the probe which had the largest mean fluorescence intensity across all samples was selected. Samples with TGF-β1 treated for 72 hours and untreated were employed for EMT-related gene identification.

Statistic analysis

EMT-related genes for both M-BE and A549 cell lines were identified using linear models and empirical Bayes' methods [5], with Benjamini and Hochberg's [6] false discovery rate (FDR) corrected *P*-value<0.01 and 2-fold changes as the significant cutoff. Significance Analysis of Microarrays (SAM) [7] was employed to assess the genes associated with lymph node metastasis in the lung SCC dataset. Gene Set Enrichment Analysis (GSEA) [8] of EMT-related genes in the lung SCC dataset was performed, using 1000 iterations of sample-shuffling. Unsupervised hierarchical clustering analysis and visualization of gene expression profile were performed using "gplots" package of R software (http://www.r-project.org).

The k-Nearest Neighbour (kNN, "class" package of R) classification method was employed to identify the gene-expression subtype of NSCLC. For each out of the 4 genes, gene expression value was transformed into *Z* value in CICAMS dataset and TCGA dataset, respectively. The training process was performed in CICAMS dataset, whose EMT-like subtype was identified by hierarchical clustering, using 5-fold cross validation (with 1000 random repeats) to choose the best k values with the lowest error rate for subtype prediction. In the test process, the EMT-related gene-expression subtype of NSMs from TCGA dataset was predicted by a kNN model with k=5, using the CICAMS dataset as the training set. The mutual relationship of samples with predicted subtype was visualized by 2-dimension scatter plotting with classical multidimensional scaling using Euclidean distance.

Pearson χ^2 test with Yates continuity correction or Fisher's exact test was performed in the association analysis between gene subtype and clinical parameters. The significance of qRT-PCR data and public EMT dataset was analyzed by unpaired Student's t-test. For those data without normal distribution (Shapiro-Wilk test, $P < 0.05$), log-transformation was performed before t-test was carried out. Kaplan-Meier curves and log-rank test were used to compare the overall survival rate of patients with different EMT-like gene-expression subtypes. Multivariate Cox proportional hazards regression model was performed. These results were analyzed using "survival" package of R.

References

1. Cai X, Xiao T, James SY, Da J, Lin D, Liu Y, Zheng Y, Zou S, Di X, Guo S, Han N, Lu YJ, Cheng S, Gao Y and Zhang K. Metastatic potential of lung squamous cell carcinoma associated with HSPC300 through its interaction with WAVE2. Lung Cancer. 2009; 65(3):299-305.

2. Hornberger J, Cosler LE and Lyman GH. Economic analysis of targeting chemotherapy using a 21-gene RT-PCR assay in lymph-node-negative, estrogen-receptor-positive, early-stage breast cancer. Am J Manag Care. 2005; 11(5):313-324.
3. Livak KJ and Schmittgen TD. Analysis of relative gene expression data using real-time quantitative PCR and the 2(-Delta Delta C(T)) Method. Methods. 2001; 25(4):402-408.
4. Feng L, Liu H, Liu Y, Lu Z, Guo G, Guo S, Zheng H, Gao Y, Cheng S, Wang J, Zhang K and Zhang Y. Power of deep sequencing and agilent microarray for gene expression profiling study. Mol Biotechnol. 2010; 45(2):101-110.
5. Smyth GK. Linear models and empirical bayes methods for assessing differential expression in microarray experiments. Stat Appl Genet Mol Biol. 2004; 3:Article3.
6. Hochberg YBaY. Controlling the false discovery rate: a practical and powerful approach to multiple testing. Journal of the Royal Statistical Society. 1995; 57:289-300.
7. Tusher VG, Tibshirani R and Chu G. Significance analysis of microarrays applied to the ionizing radiation response. Proc Natl Acad Sci U S A. 2001; 98(9):5116-5121.
8. Subramanian A, Tamayo P, Mootha VK, Mukherjee S, Ebert BL, Gillette MA, Paulovich A, Pomeroy SL, Golub TR, Lander ES and Mesirov JP. Gene set enrichment analysis: a knowledge-based approach for interpreting genome-wide expression profiles. Proc Natl Acad Sci U S A. 2005; 102(43):15545-15550.

Supplementary Tables

Supplementary Table S1. Comparison of clinical phenotype of two lung SCC datasets

[1]Variables		Lung SCC cohorts		
		discovering(%)	validation(%)	[2]Pvalue
Gender				0.699651†
	Female	3(5)	4(8)	
	Male	57(95)	46(92)	
Age				0.355891
	<65ys	34(56.7)	23(46)	
	≥65ys	26(43.3)	27(54)	
Smoking				0.905572
	<20pys	13(21.7)	8(16)	
	≥20pys	44(73.3)	29(58)	
	N/A	3(5)	13(26)	
T Stage				0.30388†
	1	3(5)	3(6)	
	2	36(60)	36(72)	
	3	17(28.3)	8(16)	
	4	4(6.7)	1(2)	
	N/A	0(0)	2(4)	
LymphNode				0.77877
	No	26(43.3)	23(46)	
	Yes	34(56.7)	25(50)	
	N/A	0(0)	2(4)	
TNM Stage				0.204573
	Ⅰ	19(31.7)	21(42)	
	Ⅱ	15(25)	14(28)	
	Ⅲ	26(43.3)	13(26)	
	N/A	0(0)	2(4)	
Tumor Differ.				0.85127†
	Well	4(6.7)	2(4)	
	Moderate	24(40)	21(42)	
	Poor	27(45)	20(40)	
	N/A	5(8.3)	7(14)	

[1] ys, years; pys, package years; Differ. , differentiation; N/A, not available. [2] †, Fisher's exact test; others, chi-square test; the missing value was not included in both tests.

Supplementary Table S2. Clinical phenotype of NSCLC cohort from TCGA

[1]Variables		Number(%)
**Pathology**		
	ADC	37(68.5)
	SCC	17(31.5)
**Gender**		
	Female	22(40.7)
	Male	32(59.3)
**Age**		
	<65ys	19(35.2)
	≥65ys	35(64.8)
**Smoking**		
	<20pys	9(16.7)
	≥20pys	30(55.5)
	N/A	15(27.8)
**T Stage**		
	1	14(25.9)
	2	35(64.8)
	3	3(5.6)
	4	1(1.9)
	N/A	1(1.9)
**LymphNode**		
	No	31(57.4)
	Yes	22(40.7)
	N/A	1(1.9)
**TNM Stage**		
	I	28(51.9)
	II	15(27.8)
	III	10(18.5)
	N/A	1(1.9)

[1] ADC, adenocarcinoma; SCC, squamous cell carcinoma; ys, years; N/A, not available.

See: Supplementary Table S3-S7 File

Supplementary Table S3. Significant genes in EMT model of M-BE (red indicates up-regulated genes in EMT-induced M-BE, blue for the down-regulated ones)
Supplementary Table S4. GO terms of biological process enrinchment analysis for 1490 up-regulated genes in EMT-induced M-BE
Supplementary Table S5. SAM Analysis for the Two-Class (LN+ vs LN-) Unpaired Case Assuming Unequal Variances
Supplementary Table S6. GO terms of biological process enrinchment analysis for 121 genes identified by SAM
Supplementary Table S7. Common genes identified in EMT model and NSMs with lymph node positive

Supplementary Table S8. Association analysis between 33 common genes and clinical parameters by GSEA

Phenotypes[1]	NES	NOM *p*-val	FWER *p*-val
Gender(Male vs Female)	-0.926	0.611	0.637
Age(≥ 60 ys vs < 60 ys)	1.115	0.418	0.564
Smoking(≥ 20 pys vs < 20 pys)	-1.409	0.096	0.179
T(T3&T4 vs T1&T2)	1.267	0.255	0.447
N(N1&N2 vs N0)	1.698	<0.001	<0.001
TNM Stage(Ⅱ&Ⅲ vs Ⅰ)	1.688	<0.001	0.001
Differ.(P vs M&W)	1.448	0.093	0.133

[1] ys, years; pys, package years; Differ., tumor differentiation grade; P, Poor; M, Moderate; W, Well.

Supplementary Table S9. Mutivariate Cox proportional hazards regression model in validation cohort from CICAMS

[1]variables	Hazard Ratio(HR)	[2]lower .95 CI	upper .95 CI	Pvalue
**Gender**	1.1	0.2	5.3	0.897
**Age**	1.4	0.5	3.7	0.465
**Stage**	2.0	1.1	3.6	0.024
**Differ.**	2.2	0.9	5.6	0.103
**4-gene**	3.7	1.2	10.8	0.019

[1]Gender: Male vs Female; Age: ≥ 65 ys vs < 65 ys; Stage: Ⅰ, Ⅱ and Ⅲ, continuous; Differ. = tumor differentiation grade: Well, Moderate and Poor, continuous; 4-gene: active vs inactive. [2]CI = confidence interval.

Supplementary Table S10. Mutivariate Cox proportional hazards regression model in NSCLC dataset from TCGA

[1]variables	Hazard Ratio(HR)	[2]lower .95 CI	upper .95 CI	Pvalue
**Gender**	1.1	0.4	2.5	0.904
**Age**	1.6	0.6	4.5	0.359
**Stage**	1.4	0.8	2.5	0.204
**Pathology**	1.1	0.4	2.7	0.861
**4-gene**	2.5	1.0	6.1	0.047

[1]Gender: Male vs Female; Age: ≥ 65 ys vs < 65 ys; Stage: Ⅰ，Ⅱand Ⅲ, continuous; Pathology: lung SCC vs lung ADC; 4-gene (prediction): active vs inactive. [2]CI = confidence interval.

Supplementary Table S11. Primers for qRT-PCR analysis by SYBR® Green method

GeneSymbol	Sense primer	Anti-sense primer
CDH1	5' GAACGCATTGCCACATACA 3'	5' CGGGCTTGTTGTCATTCTG 3'
CDH2	5' TCCTCCAGAGTTTACTGCC 3'	5' GTGACTAACCCGTCGTTG 3'
VIM	5' ATTCACTCCCTCTGGTTG 3'	5' TGATGCTGAGAAGTTTCG 3'
FBN1	5' ACCTGGTTACTTCCGCATAG 3'	5' TGGAGGCATCAGTTTCGT 3'
ECM1	5' TGAAGACCCACCACCACT 3'	5' AGCCCAGGAATATGTTTAT 3'
MAP1B	5' CTCCCGATTTCCTACTTA 3'	5' ATGTTGTACTGGGTGCTG 3'
LTBP1	5' CTGTGCCTGTTGAAGTAGC 3'	5' TGAACCTGTAGCCCTCGTA 3'
18S	5' TGCATGGCCGTTCTTAGTTG 3'	5' AGTTAGCATGCCAGAGTCTCGTT 3'

Supplementary Table S12. TaqMan® assay ids for qRT-PCR analysis

GeneSymbol	TaqMan ID
FBN1	Hs00973198_m1
ECM1	Hs00189441_m1
MAP1B	Hs01067018_m1
LTBP1	Hs01586499_m1
RPLP0	Hs00420895_gH
GUSB	Hs99999908_m1

Supplementary Figures

100× 400×

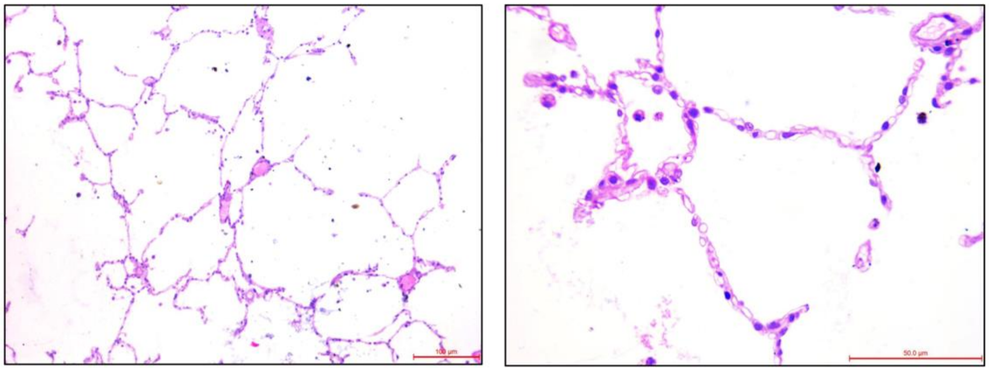

Supplementary Figure S1. Hematoxylin and eosin (H&E)-staining for NSMs of lung SCC. Formalin-fixed and paraffin-embedded tissues were used for cutting 5-μm-thick sections. Sections were deparaffinized, ethanol-rehydrated, and stained with H&E for microscopic examination. Images were photographed at 100×(left, scale bar = 100μm) and 400× (right, scale bar = 50μm) magnification using white light microscopy, respectively.

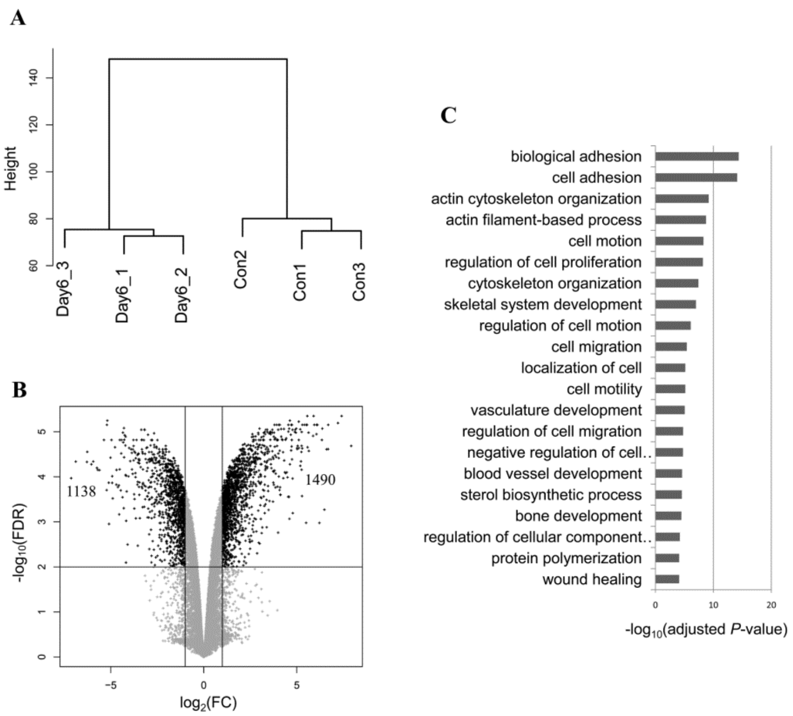

Supplementary Figure S2. Gene expression profiling analysis of the EMT model. **A**, unsupervised hierarchical clustering analysis of TGF-β1-induced (Day6_1-3) and control (Con1-3) M-BE samples, using the global gene expression data. **B**, significance analysis of the EMT model. Linear models and empirical Bayes methods were performed to estimate *P*-value. FDR was calculated using Benjamini and Hochberg's method. Each star represents one gene, FC > 2 or < 0.5 (x-axis), and FDR < 0.01 (y-axis) are set as thresholds of significance (black). **C**, GO terms of biological process enrichment analysis for 1490 up-regulated genes in B. The x-axis indicates $-\log_{10}$ transformed Benjamini-Hochberg adjusted *P*-value.

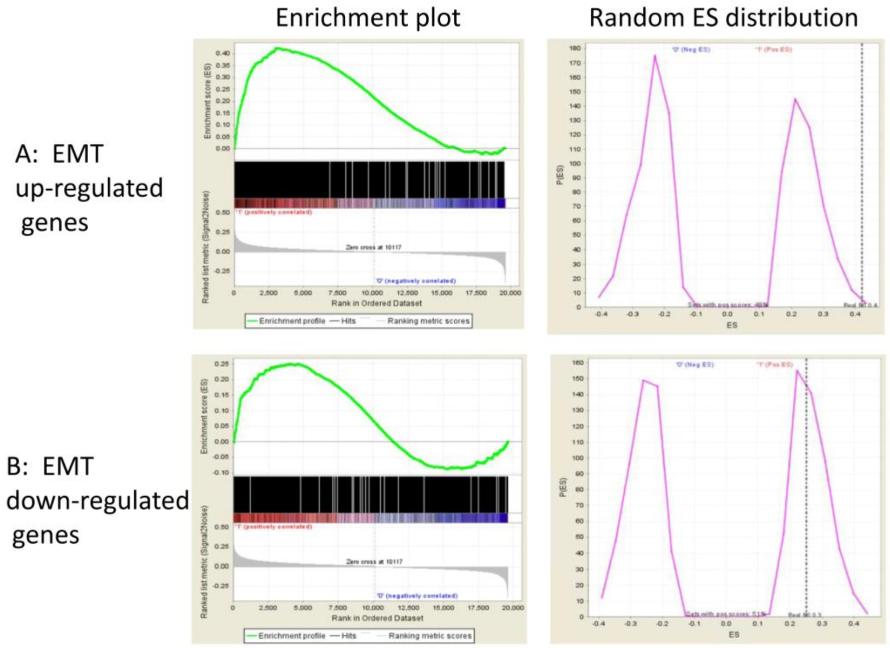

Supplementary Figure S3. Gene Set Enrichment Analysis (GSEA) of EMT-derived gene sets with lymph node metastasis in NSMs dataset. GSEA was completed with weighted enrichment statistics and 1000 iterations of sample-shuffling. Label “1” indicates the lymph node positive class, label “0” for the negative. Left panels indicate Enrichment score (ES) plotting, right panels indicate distribution of random ES derived by sample-shuffling and the real ES. **A**, EMT up-regulated gene set. **B**, EMT down-regulated gene set.

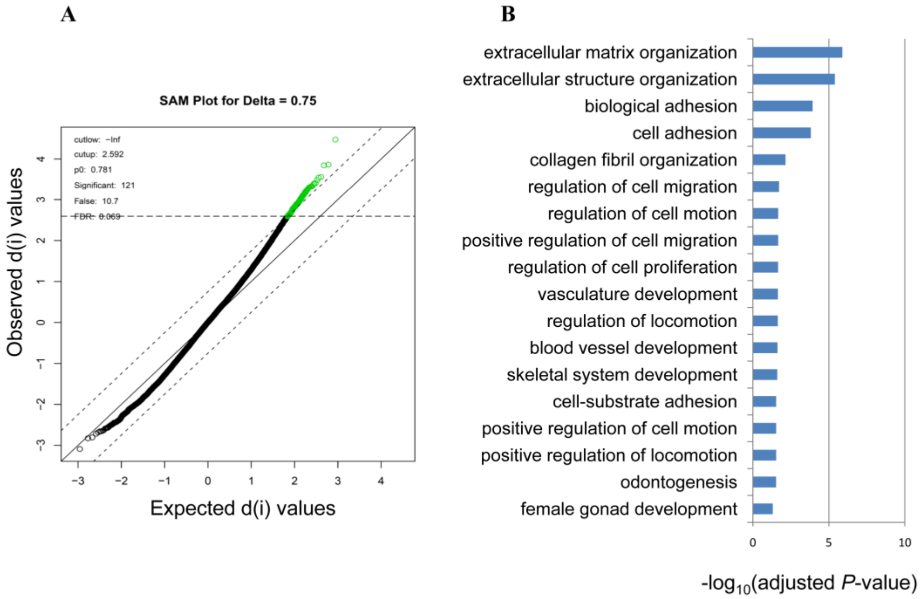

Supplementary Figure S4. Differentially expressed genes in NSMs associated with lymph node metastasis. **A**, Significance Analysis of Microarrays (SAM) for lymph node metastasis status in the noncancerous lung tissue dataset. According to the cutoff (delta = 0.75, FDR = 0.069), 121 genes (green points) were significantly up-regulated in lymph node positive samples. **B**, GO terms of biological process enrichment analysis for SAM genes in (A). X-axis indicates $\log_{10}$-transformed Benjamini-Hochberg adjusted *P*-value.

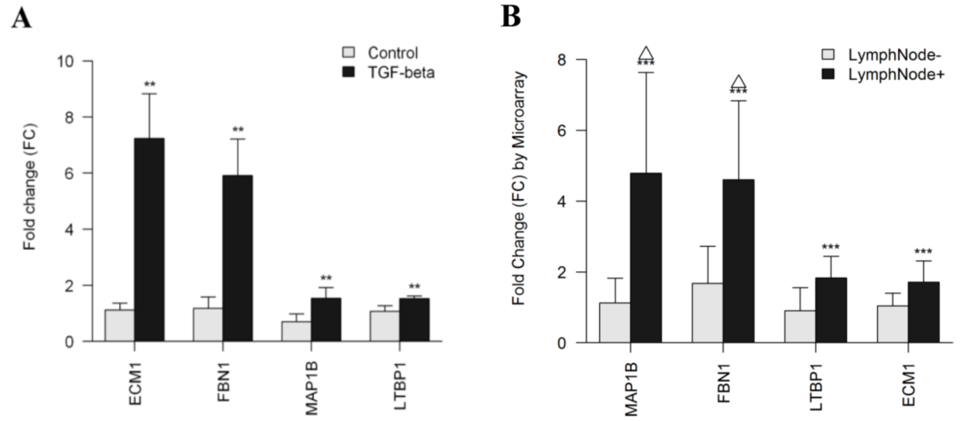

Supplementary Figure S5. Confirmation of EMT-related gene-expression profile in EMT model and NSMs of lung SCC. **A**, qRT-PCR analysis of four selected genes (*FBN1*, *ECM1*, *MAP1B*, *LTBP1*) in the EMT model of M-BE. Y-axis indicates the relative expression level (fold change) of genes. Means and standard deviations (SD, error bars) are shown. Unpaired Student's t-test (two sided) was performed for significance estimate. **M-BE cells treated with TGF-β1vs control, $P < 0.05$. **B**, qRT-PCR analysis of four selected genes in 30 NSMs of lung SCC. Y-axis indicates the relative expression level (fold change) of genes. Means and standard deviations (SD, error bars) are shown. Unpaired Student's t-test (two sided) was performed for lymph node status. Δ This group of data is not normally distributed (Shapiro-Wilk test, $P < 0.05$), and performed with log-transformation before t-test. ***Lymph node positive vs negative, $P < 0.001$.

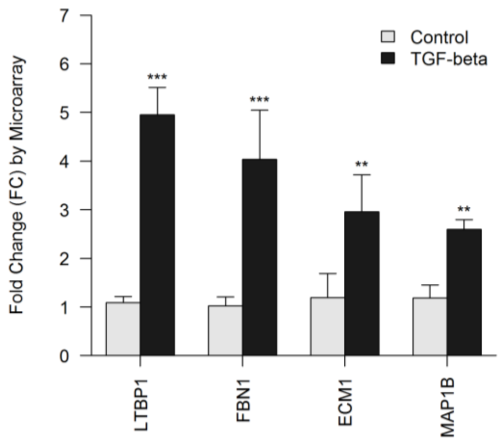

Supplementary Figure S6. Confirmation of EMT-related gene-expression profile in EMT model of A549. Gene expression profile of A549 cells underwent EMT or not was downloaded from Gene Expression Omnibus (GSE17708). Y-axis indicates the relative expression level (fold change by microarray data) of genes. Means and standard deviations (SD, error bars) are shown. Unpaired Student's t-test (two sided) was performed for significance estimate. A549 cells treated with TGF-β1 (72 h) vs control, **: $P < 0.05$, ***: $P < 0.01$.

A

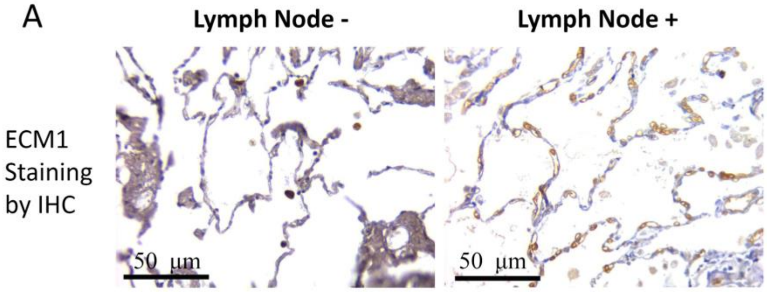

B

	ECM1 staining (IHC)	
	Negative	Positive
**Lymph Node -**	8	5
**Lymph Node +**	5	12

Fisher's Exact Test:
P = 0.138

Supplementary Figure S7. Immunohistochemical staining of ECM1 in NSMs from lung SCC. A, Formalin-fixed and paraffin-embedded tissues were used for cutting 5-μm-thick sections. A negative staining in lymph node negative NSM sample was shown in left, and a positive one with lymph node metastasis NSM in the right (400×, scale bar = 50μm). B, Statistic of ECM1 staining and lymph node status in 30 NSMs of lung SCC from the discovering cohort.

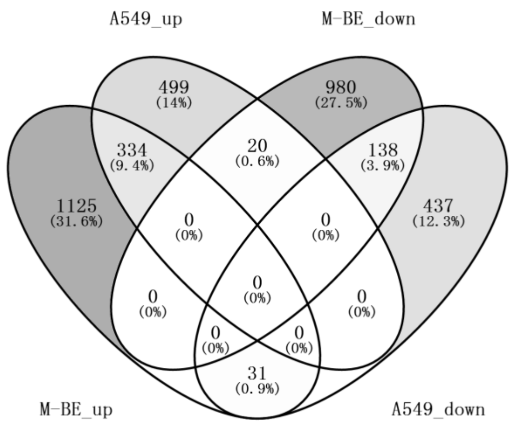

Supplementary Figure S8. The overlap of gene sets from EMT models of two cell lines. Gene expression profile of EMT model of A549 was downloaded from Gene Expression Omnibus (GSE17708). The up-regulation (A549_up) and down-regulated (A549_down) genes were derived from comparing A549 cells with TGF-β1-treatment for 3 days to control cells. For both M-BE and A549, FDR < 0.01 and 2-fold changes were set as the significant cutoff. The significance of overlap between two gene sets was estimated by hypergeometric test. The overlaps between gene sets with the same directions (M-BE_up and A549_up, $P < 0.001$; M-BE_down and A549_down, $P < 0.001$) were significant, while the gene sets with opposite direction (M-BE_up and A549_down, $P = 0.99$; M-BE_down and A549_up, $P = 0.998$) did not.
